# A Report on the Progress of Obstetrics and of Gynæcology

**Published:** 1874-02-15

**Authors:** William Goodell


### Gleanings from Our Exchanges.

### A REPORT ON THE PROGRESS OF OBSTETRICS AND OF GYNÆCOLOGY.

By William Goodell, M.D.

*From Transactions of Medical Society of State of Pennsylvania*, 1873.

The Abortive Action of Quinia.—The thrice-vexed question of the action of quinia upon the uterus has claimed a large share of attention. That this agent does sensibly excite uterine contractions can hardly be doubted; the evidence on this point is overwhelming. Yet it is uncertain whether the few reported cases of abortion under its use have been owing to this action, or to the paludal poison for which it was prescribed. The testimony here is so conflicting, that the *Societe de Medecine*, of Gand, in Belgium, has proposed the subject as a prize essay for 1874. After carefully weighing the evidence of his own experience and that of others, your reporter has arrived at the following conclusions: 1. That quinia, by producing intermittent contractions of the womb, has, in large doses, occasionally brought on an abortion in the very early months of gestation. 2. That it should not on that account, however, be withheld from pregnant ague-patients; for, other things being equal, an abortion is more likely to be induced by the visceral congestions and muscular succussions attending an attack of ague than by the oxytocic property of the antiperiodic. 3. That the uterine action of this drug is too slow and too uncertain to be relied upon in the emergencies of ante or post-partum hæmorrhages. But that, in decided doses, it will often prove of service in menorrhagic or metrorrhagic attacks. 4. That, like ergot, it acts most efficiently after labor has begun; a dose of ten grains being usually followed, in inertia, by a prompt return of the pains. 5. That, apart from its tonic and antiseptic properties, quinia is *par excellence* the remedy for puerperal disorders. By lowering the high temperature generated both by accelerated molecular metamorphosis and by rapid chemical combinations, it retards the oxidation of the tissues, hinders the formation of fibrinous concretions, and, therefore, prevents cardiac plugging. By contracting the walls of the womb, it tends to keep the protective coagula of the uterine sinuses from becoming loose and soluble, and to inhibit putrid and purulent absorption. Both by constringing the coats of the capillary system of blood-vessels, and by paralyzing the amœboid movement of the white blood-corpuscles, it presents, in puerperal fevers, an obstacle to fibrinous exudation and to the migration of the leucocytes into serous cavities.

The Delivery of the Placenta by Supra-Pubic Pressure.—Judging from our own experience and from the number of laudatory papers on this subject, Crede's method of delivering the placenta, or some slight modification of it, bids fair to take the place of every other. The plan which we adopt is as follows: At the maximum of the first uterine contraction after the birth of the child, the fundus of the womb is grasped, through the abdominal wall, between the thumb in front and the fingers behind. It is then to be both forcibly squeezed and at the same time pressed downward and backward. By means of this uterine expression the placenta and membranes are usually at once detached and extruded. Sometimes, indeed, they will suddenly pop out of the vulva, just as the stone escapes when a cherry is compressed between the finger and thumb. Occasionally it will require two or more pains to effect this; but the sooner this plan is resorted to after the birth of the child, the more easy in execution will it be. Those who, like ourselves, practice this method, contend that it offers many advantages over any other. The risk of communicating any puerperal disease is lessened. The expulsion of the placenta and membranes by a *vis a tergo* is more likely to be complete, than by traction on the cord. The cord cannot be broken, as no traction is made on it. Adherent placenta is less frequently met with. The introduction of the hand into the womb is avoided, and so, also, as a consequence, is the ingress of air. Finally, the tonic and energetic contraction of the womb, following this manœuvre, prevents the occurrence of hæmorrhage or of unruly after-pains.

*Bibliography.*—*Amer. Jour. of Obstetrics*, Aug., 1871, p. 334; Transactions of the Indiana State Medical Society for 1871; Schroeder's Obstetrics, 1872; Medical Correspondenz-Blatt, 10, 1873; Lo Sperimentale, April, 1873.

PUERPERAL ECLAMPSIA. — By its property of diminishing the tension of the blood-vessels, and by thus relieving the intra-cerebral pressure, the nitrite of amyl bids fair to prove a valuable addition to our means for treating puerperal eclampsia. Dr. W. F. Jenks (*Philadelphia Med. Times*, Aug. 1, 1872, p. 404) reports a case in which, after two violent convulsions, he, at the suggestion of Dr. S. Weir Mitchell, administered by inhalation two or three drops of this agent, "when the premonitory twitching, the contracted pupils, and the convergent strabismus announced the return of a seizure. The effect was magical: the muscles relaxed, the strabismus disappeared, the face flushed, and the patient remained quiet for a longer or shorter time." Its use is, however, apparently attended by a partial paralysis of involuntary muscular fibre, for in the reported case a profuse post-partum hæmorrhage took place, calling for a uterine injection of a weak solution of the subsulphate of iron. This tendency to post-partum hæmorrhage we have, however, repeatedly seen in eclampsia. In another case, treated by the same gentleman (*American Supplement to the Obstetrical Journal of Great Britain*, April, 1873, p. 3), he hesitated, on account of this property, to resort to the nitrite of amyl. He was, however, successful by bleeding and by drastic cathartics.

The treatment of puerperal eclampsia still remains unsettled. The profession is divided into those who deem this disease to be caused by serous apoplexy, and those who attribute it to uræmic poisoning, or to nervous exhaustion — into those, consequently, who bleed and those who do not. The latter have, hitherto, had the large majority; but the signs of a reaction are manifest. There is, evidently, a growing tendency, first, to lessen provisionally the intravascular pressure by an early and full bleeding, before resorting to anæsthesia, narcotics, and the drastic cathartics. Upon the great value of the hydrate of chloral in controlling the convulsive attacks, we forbear to enlarge. This agent has so generally received the encomiums of the profession that it is needless for us to do more than to advert to its use, and we, therefore, subjoin but one reference (*Lancet*, April 12, 1873). With this remedy we like to combine the bromide of potassium in full doses. In the treatment of this disease it often becomes a very nice point to determine whether or not labor should be either induced or urged on. To decide this grave question, your reporter would diffidently suggest the following broad rules of guidance: If the convulsions are uncontrollable or the woman is near to term, if the os has begun to dilate, or the face is œdematous and the urine loaded with albumen, then, as the case may be, either induce labor or hasten it on. Should these conditions not be present, the indication will be to avoid exciting the uterus to premature action. Either in inducing a premature labor, or in hastening on a labor already begun, your reporter has found nothing better than the hydrostatic bags of Dr. Barnes — the patient being kept profoundly anæsthetized.

*Bibliography*.—Fordyce Barker, *New York Med. Jour.*, Jan., 1871, p. 1; B. W. Richardson, *London Practitioner*, Nov., 1868, p. 274; W. F. Jenks, *Philadelphia Med. Times*, May 1, 1871, p. 285; A. W. Tupper, *Transactions of the New York State Med. Society*, 1872, p. 216; Joseph Carson, *Amer. Jour. of the Med. Sciences*, vol. lxi., 1870, p. 433; A. B. Steele, *Med. Times and Gazette*, Aug. 24, 1872; E. Montgomery, *St. Louis Med. and Surg. Jour.*, Sept., 1872; R. Barnes, *Lancet*, April and May, 1873, pp. 516, 619.

